# Food pattern modeling shows that the 2010 Dietary Guidelines for sodium and potassium cannot be met simultaneously

**DOI:** 10.1016/j.nutres.2013.01.004

**Published:** 2013-03

**Authors:** Matthieu Maillot, Pablo Monsivais, Adam Drewnowski

**Affiliations:** a Aix Marseille Université, NORT, 13005, Marseille, France; b Inserm, UMR_S 1062, 13005, Marseille, France; c Inra, UMR_INRA 1260, 13005, Marseille, France; d Centre for Diet and Activity Research, Box 296, Institute of Public Health Forvie Site, Robinson Way, Cambridge CB2 0SR; e Nutritional Sciences Program and Center for Public Health Nutrition, School of Public Health, University of Washington, Seattle, WA 98195-3410

**Keywords:** Linear programming, Sodium, Potassium, Dietary guidelines, Nutrient adequacy, DRIs, Dietary Reference Intakes, FNDDS, Food and Nutrition Database for Dietary Studies, LP, linear programming, NHANES, National Health and Nutrition Examination Survey, USDA, United States Department of Agriculture

## Abstract

The 2010 US Dietary Guidelines recommended limiting intake of sodium to 1500 mg/d for people older than 50 years, African Americans, and those suffering from chronic disease. The guidelines recommended that all other people consume less than 2300 mg sodium and 4700 mg of potassium per day. The theoretical feasibility of meeting the sodium and potassium guidelines while simultaneously maintaining nutritional adequacy of the diet was tested using food pattern modeling based on linear programming. Dietary data from the National Health and Nutrition Examination Survey 2001-2002 were used to create optimized food patterns for 6 age-sex groups. Linear programming models determined the boundary conditions for the potassium and sodium content of the modeled food patterns that would also be compatible with other nutrient goals. Linear programming models also sought to determine the amounts of sodium and potassium that both would be consistent with the ratio of Na to K of 0.49 and would cause the least deviation from the existing food habits. The 6 sets of food patterns were created before and after an across-the-board 10% reduction in sodium content of all foods in the Food and Nutrition Database for Dietary Studies. Modeling analyses showed that the 2010 Dietary Guidelines for sodium were incompatible with potassium guidelines and with nutritionally adequate diets, even after reducing the sodium content of all US foods by 10%. Feasibility studies should precede or accompany the issuing of dietary guidelines to the public.

## 1 Introduction

Americans consume too much sodium and not enough potassium. The high ratio of sodium to potassium (Na-K) has been linked to high blood pressure, heart disease, and stroke [1-4]. In recent analyses of the National Health and Nutrition Examination Survey (NHANES) III (1988-1994) mortality data, higher ratios of Na-K were independently linked to a higher risk of cardiovascular diseases and to higher all-cause mortality [5].

**Fig. 1:**
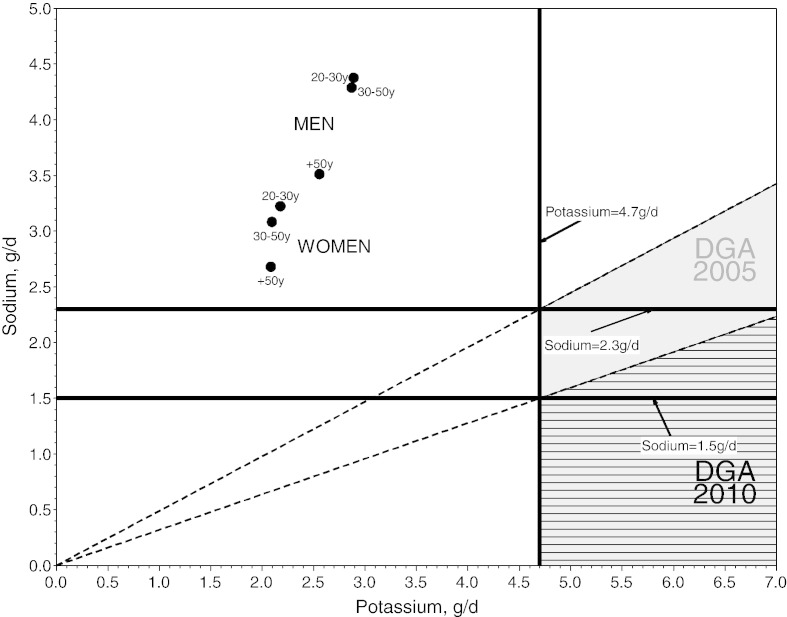
The recommended values for the consumption of sodium and potassium as listed in the 2005 and 2010 Dietary Guidelines compared with actual intakes for 6 age-sex groups in the 2001-2002 NHANES database. Footnote: The gray area represents compliance with 2005 Dietary Guidelines for Americans (DGAs); the hatched area is the 2010 DGAs.

Public health recommendations have stressed that a reduction in sodium intake should be accompanied by a simultaneous increase in potassium intake. The net effect would be to reduce the ratio of Na-K in the American diet. The 2010 Dietary Guidelines [6] advised Americans to reduce daily sodium intake to less than 2300 mg/d per person, with an even lower goal of 1500 mg/d set for persons who were 51 years and older; were African American; or suffered from hypertension, diabetes, or kidney disease. The potassium goals remained at 4700 mg/d (Fig. 1). To meet such goals, the dietary ratio of Na-K would need to be in the order of 0.49 (2300/4700).

Both sodium and potassium intakes are closely related to total energy intakes [7]. Current sodium consumption in the United States is estimated at 2300 to 4500 mg/d depending upon sex and age, whereas current potassium consumption varies from 2400 to 3200 mg/d [8]. A reduction of sodium intakes by two-thirds while doubling potassium intakes may require some dramatic shifts in eating habits. Arguably, persons with low energy intakes may successfully meet the reduced sodium goals but will not reach adequate potassium levels. By contrast, persons with high energy intakes may achieve potassium goals but will fail to meet the low guidelines for sodium. Food pattern modeling, based on linear or nonlinear programming [9-13], has been used to create food patterns that comply with dietary recommendations while taking existing eating habits into account [14]. Mathematical modeling of food patterns can also help determine whether an across-the-board 10% reduction in the sodium content of the US food supply would make the sodium and potassium goals easier to meet.

The 2010 Dietary Guidelines called for a broad-based reduction in the sodium content of foods to allow for the sodium goals to be met. The present study used linear programming (LP) to determine the feasibility of meeting the sodium and potassium guidelines in the context of nutritionally adequate diets. Food patterns that were consistent with the recommended ratio of Na-K (2300/4700 or 0.49) and caused the least deviation from the existing food habits were also identified.

## 2 Methods and materials

### 2.1 Dietary data

Observed diets for 6 groups by sex and age were based on the 2001-2002 NHANES sample of 4295 individuals. Pregnant women and persons with reported daily intakes of less than 600 kcal/d were excluded [15]. Nutritionally adequate food patterns were developed for men and women in 3 age groups, as follows: 20 to 30, 31 to 50, and more than 50 years.

A modeled food pattern was created using LP models for each sex-age group. An LP model is defined by a list of food variables, a list of constraints (nutritional and behavioral constraints), and an objective function. In this study, the list of food variables was provided by dietary intake data from NHANES. Nutritional composition data were provided by the Food and Nutrition Database for Dietary Studies (FNDDS 1.0) from the US Department of Agriculture (USDA). The nutritional constraints used in the LP model were based on Dietary Reference Intakes (DRIs) for 26 nutrients including both potassium and sodium. The consumption constraints were based on the observed distribution of consumption of food groups and food categories in the NHANES databases. All these input data varied depending on the sex-age groups and were described in detail in 2 previous articles [16,17].

### 2.2 Description of LP modeling procedures

#### 2.2.1 Food variables

Only foods that were consumed by participants in the 2001-2002 NHANES were entered into food groupings. Baby foods, alcohol, medical foods and supplements, electrolyte solutions, chewing gum, and foods with an energy density less than 10 kcal/100 g (eg, water, coffee, tea) were excluded. For modeling purposes, individual foods consumed by each age-sex group were collapsed into food categories that represented the food variables in each LP model. The number of food categories (ie, food variables) by age-sex subgroups varied from 85 to 128. The nutrient profile of each food category was calculated based on a weighted average of the nutrient contribution of each food [16,17], as based on the FNDDS 1.0 nutrient composition data [18] and frequency of occurrence in the 2001-2002 NHANES [12,13,19,20]. The corresponding values for solid fat and added sugar in grams and calories were obtained from the *MyPyramid Equivalents Database for USDA Survey Food Codes Version 1.0* [21].

#### 2.2.2 Energy and nutritional constraints

The energy constraint for each group was set equal to the energy requirement for that group [17]. Estimated energy requirements for each sex-age group were based on previous work by the Institute of Medicine [22] and USDA [23]. Nutritional goals or constraints ensured the nutritional adequacy of each food plan and its compliance with Dietary Guidelines. Population-wide standards were used for protein, total carbohydrates, total lipids, saturated fatty acids, linoleic acid, linolenic acid, cholesterol, and added sugar [22]. Age- and sex-specific recommended dietary allowances values of DRI [24-27] were used for fiber; vitamins A, C, and E; thiamin; riboflavin; niacin; vitamin B6; folate; and vitamin B12 and for calcium, copper, iron, magnesium, phosphorus, selenium, and zinc. Because men aged 20 to 30 years did not report consumption of vegetable oils, the constraint for vitamin E was reset to 13 mg/d, assuming a consumption of 1 tablespoon of vegetable oil per day (ie, 2 mg/d of vitamin E) [17].

#### 2.2.3 Food group constraints

The food categories (ie, food variables) were collapsed into 39 food subgroups and 9 major food groups. The groupings largely followed the USDA system established for coding foods and amounts in “What We Eat in America/NHANES 2001-2.” The number of food subgroups varied from 35 to 39 depending on the sex-age group. The 9 major food groups have been described elsewhere [17].

Sex- and age-specific consumption constraints were placed on food groups and subgroups and on each food category. Modeled amounts for the 9 food groups were bounded by the 10th and 90th percentiles of the observed consumption in the referent sex-age group. Upper bounds for food subgroups and food categories were set by the 75th percentile of observed consumption of the referent age-sex group [17].

#### 2.2.4 Objective functions: maximizing potassium and minimizing deviation from eating habits

Two different objective functions were used. The first objective function maximized the total amount of potassium in the optimized food patterns. For each sex-age group, LP models created food patterns that maximized the amount of potassium in a nutritionally adequate food pattern while holding the ratio of Na-K constant at 0.49 (2300/4700). The objective was to determine the boundary conditions for the potassium and sodium content of the optimized food patterns that would be compatible with nutrient adequacy. This function helped define the mathematically feasible area for potassium and sodium combinations. In the second set of models, the ratio of Na-K constraint was removed. Linear programming models now sought to maximize potassium as the permitted sodium content of the modeled food patterns was progressively reduced. Starting from the amount of sodium found in the first step, the sodium amount fixed in optimized food patterns was decreased by 0.1 g until the LP model became infeasible.

The second objective function minimized the deviation between the modeled food patterns and the existing food habits [10,14,17,28,29]. This model held the ratio of Na-K constant but allowed the sodium or potassium content of the modeled food patterns to vary. The optimized food patterns yielded the combinations of potassium and sodium that were compatible with nutrient-adequate diets (DRIs) while showing the least deviation from the observed eating habits.

### 2.3 Simulating reduction in sodium content of food supply

The LP models described above were tested before and after a simulated across-the-board 10% reduction of sodium content of all foods in the FNDDS 1.0 nutrient composition database. For each sex age-group, new boundary conditions for feasible Na-K combinations that were compatible with nutritionally adequate food patterns were determined. These combinations were nutritionally adequate.

## 3 Results

### 3.1 The feasibility of meeting sodium and potassium guidelines

**Fig. 2:**
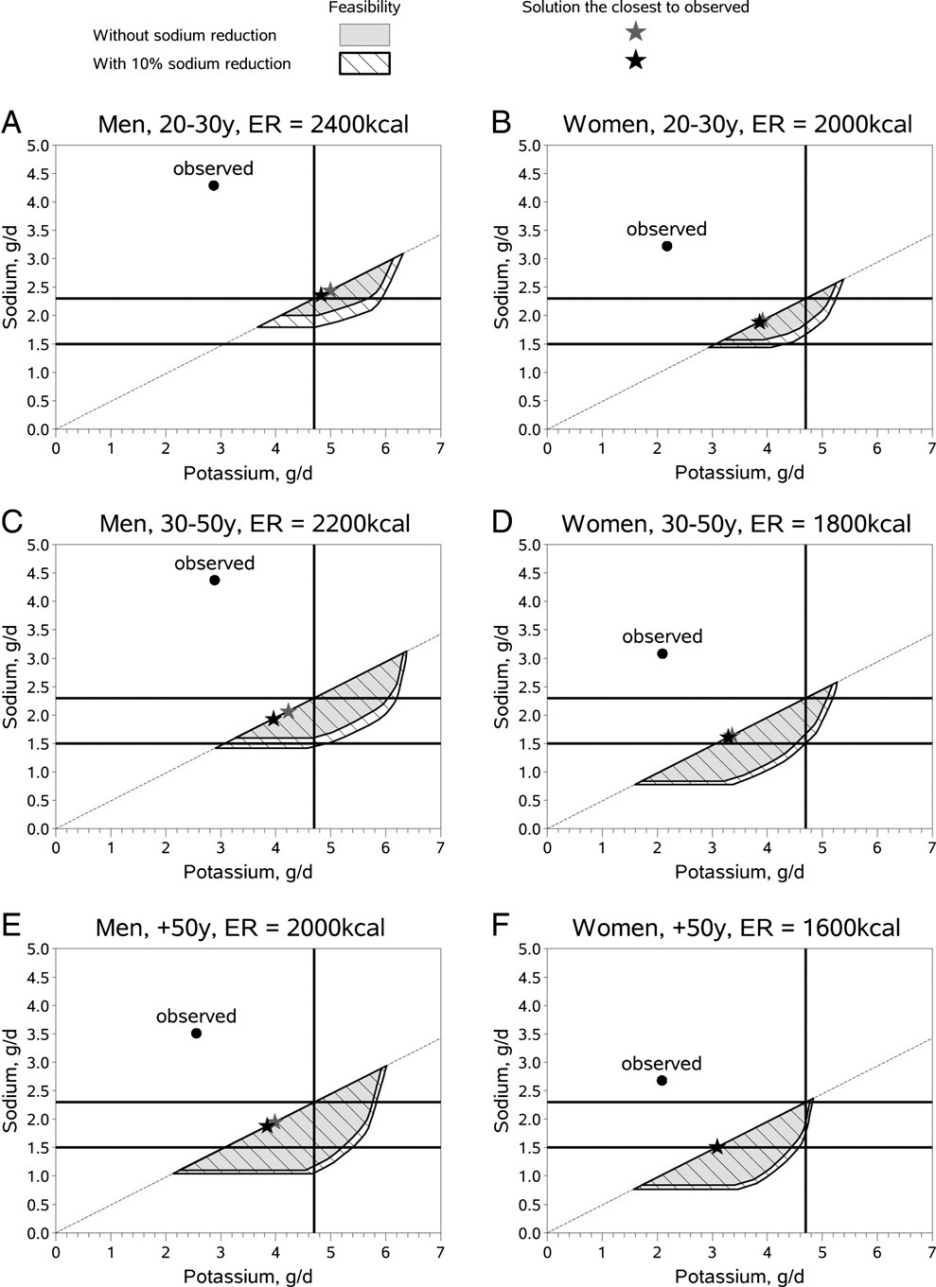
All combinations of potassium and sodium that are compatible with the fulfillment of a whole set of nutrient recommendations using the actual US food supply and assuming a 10% sodium reduction across the board for 6 sex-age groups.

Using a series of LP models, the allowable ranges of sodium and potassium in food patterns that met the ratio of Na-K constraint of 0.49 and also met all nutrient recommendations for the 6 sex-age groups were identified and plotted as feasibility areas. They are presented in Fig. 2 as the shaded, hatched area in each plot.

The 2300-mg/d sodium guideline was theoretically compatible with the potassium guideline (4700 mg/d) for all sex-age groups (illustrated by the portion of the feasibility areas at or below the 2.3-g/d sodium line and at or beyond the 4.7-g/d potassium line). Food patterns for men older than 30 years met the sodium and the potassium goals, whereas women older than 50 years could reach the 4700-mg/d potassium goal only at the maximum level of sodium. For the remaining women and younger men, only a few Na-K combinations were compatible with nutritionally adequate diets; however, these sodium (2300 mg/d) and potassium (4700 mg/d) guidelines were feasible for some population subgroups.

The 1500-mg/d sodium guideline was theoretically compatible with the potassium guideline (4700 mg/d) only for men older than 50 years and no other group (ie, only for this group did part of the feasibility area lie below the 1.5-g/d sodium limit and extend to the 4.7-g/d potassium guideline). In such modeled food patterns, deviation from observed diets was large in terms of total weight and food choices. In general, the optimized food pattern demonstrated more servings of fruits, vegetables, and whole grains, and fewer servings of refined grains and meats (Table). Furthermore, the number of servings for milk and milk products was also higher in this modeled pattern. However, even for older men with lower energy intakes, the theoretically feasible Na-K combinations were limited.

### 3.2 The impact of reducing sodium content of the food supply by 10%

The food patterns for 6 age-sex groups were recalculated after the sodium content of all foods in the FNDDS database was reduced across-the-board by 10%. The feasibility areas reflecting this change are plotted in Fig. 2 as the combined shaded and unshaded hatched areas. This analysis showed that an across-the-board 10% reduction in the sodium content of the food supply had a minimal impact on the low energy food patterns. The feasibility of reaching the sodium goals by women was not affected.

By contrast, the reduction in sodium content of the food supply led to food patterns for men that were significantly reduced in sodium content; however, the sodium and potassium guidelines could not be met simultaneously. That particular joint goal was not feasible, and no mathematical solution was obtained.

### 3.3 Minimizing deviation from existing eating habits

For young men (ages 20-30 years), optimized food patterns compatible with all nutrient goals and with a ratio of Na-K equal to 0.49 contained 5 g/d of potassium and 2.5 g/d of sodium. For all other sex-age groups, the nutritionally adequate food patterns that were closest to existing food habits and had a ratio of Na-K equal to 0.49 had to contain 1.5 to 2.3 g/d of sodium and 3 to 4.7 g/d of potassium. Although a food pattern containing exactly 1500 mg/d sodium and 4700 mg/d potassium could be constructed for men older than 50 years, such a pattern entailed a larger deviation from existing food habits (Table).

## 4 Discussion

Previous modeling studies have pointed to the challenges of creating food patterns that were nutritionally adequate and still able to meet the sodium and potassium goals [10,13,19,29,30]. The potassium goal was more difficult to reach at low energy intakes [16,19], whereas the sodium goal was more difficult to reach at high energy intakes [13,19].

This study used LP techniques to determine the amounts of sodium and potassium that would be compatible with nutritionally adequate diets at a fixed ratio of Na-K (0.49). The nutritional and consumption constraints used in the models were similar to those used in previous studies and have been published [16,17]. The ratio of Na-K has not been modeled before.

The current sodium (2300 mg/d) and potassium goals (4700 mg/d) were, in theory, jointly feasible for all sex-age groups. By contrast, the more restrictive 1500-mg/d sodium goal was compatible with the potassium goal and other nutrient goals only for men older than 51 years.

As expected, food patterns created for young men with high energy requirements had adequate potassium but failed to meet sodium targets. By contrast, food patterns developed for older women with low energy requirements met the low-sodium goal but failed to provide adequate potassium. These modeling analyses suggest that, given the current food supply, nutrient-adequate patterns cannot go below a certain minimum level of sodium. If the level of sodium is set too low, the other nutrient requirements cannot be met; hence, the sodium goal becomes incompatible with nutrient-adequate diets. At the very least, some consideration should be given to adjusting the sodium goals to energy requirements of different population groups.

The DRIs established the minimally adequate sodium amount at 1500 mg/d based on physiologic evidence [27]. In practice, nutritionally adequate food patterns that comply with multiple dietary guidelines may require a minimum level of sodium. For example, several previous studies [10,13,19,29] have shown that dietary recommendations for fiber and many other nutrients can be achieved through increased consumption of whole grains, vegetables, and beans. However, these foods also contain sodium, sometimes added during cooking [18], and are therefore excluded from the low-sodium food patterns. For persons with energy intakes in the 2000- to 2400-kcal/d range, the overly low sodium goal of 1500 mg/d is wholly incompatible with nutrient-adequate diets.

The maximal amount of potassium in modeled food patterns was driven by the total energy requirements and by the need to meet multiple nutrient recommendations, including sodium. As expected, the goal of 4700 mg/d of potassium was difficult to reach at low energy requirements. Not surprisingly, previous studies on diet optimization have “relaxed” the potassium requirement by 10%, perhaps because it was impossible to meet [12,19].

The potassium goal of 4700 mg/d per person may also need to be revised to take differential energy needs into account. For example, in the Dietary Approaches to Stop Hypertension trial, potassium levels in the Dietary Approaches to Stop Hypertension (combination) food pattern were adjusted for energy, with 4673 mg K in a 2100-kcal diet [31]. At low energy levels, the modeled food patterns found that the potassium goal was not compatible with the fulfillment of DRIs.

The observed ratio of Na-K, approximately constant at different levels of energy intake, was 1.2. In other words, using a ratio of Na-K recommendation (0.5) would allow keeping a balance between potassium and sodium even if the potassium goal is not reached at low energy levels or sodium limits are exceeded at high energy levels.

In the present food patterns, the Na-K ratio was kept constant at 0.5. When this ratio was the only constraint (but not the absolute limits on sodium or minimum for potassium), complying with the ratio of Na-K would have been feasible for every age-sex group. Optimized food patterns with a fixed ratio of Na-K = 0.5 were closest to the observed food habits. In other words, it was easier to achieve the desired ratio of Na-K than to achieve the absolute amounts of sodium and potassium in the diet. For most food patterns, except those at the highest levels of energy intake, the ratio of Na-K of 0.5 was achieved with amounts of potassium lower than 4.7 g/d and amounts of sodium lower than 2.3 g/d. At the higher energy level, the optimal combination was 5 g/d potassium and 2.5 g/d sodium. Arguably, the optimal ratio of Na-K might replace separate dietary guidelines for potassium and sodium.

Another way to ensure compliance with the sodium and potassium guidelines is to alter the nature of the food supply. The 2010 Dietary Guidelines recommended that the sodium content of the US food supply be reduced gradually to allow consumers time to accept the taste of lower-sodium foods. The present LP models accordingly tested whether the sodium and potassium guidelines would be easier to achieve once the sodium content of the food supply were reduced across the board by 10%. However, that manipulation proved to be ineffective.

This study had limitations. First, because the FNDDS 1.0 database lacked vitamin D, the optimized food patterns did not include the vitamin D recommendation. Achieving the adequate intake for vitamin D (ie, 5 μg/d) in the optimized food patterns will likely require more milk and more fish than estimated by the present model. Because milk also contains sodium, the minimum amount of sodium may be underestimated. Second, the feasibility area for the permitted amounts of sodium and potassium in the optimized food patterns was a function of the LP model and its constraints. One such constraint was that the LP model was based on composite food categories and not on individual foods. A larger variety of individual foods may have allowed for larger feasibility areas and more combinations of individual foods that were compatible with the sodium and potassium goals. However, the permitted amounts of potassium and sodium were more strongly influenced by the nutrient and consumption constraints than by the number of food categories, which ranged from 62 to 120. The number was adequate, given that a typical person consumes approximately 40 different foods per week [14]. The nutrient composition of food categories as consumed was much more realistic than that of the idealized MyPyramid food groups [32]. Another limitation related to food pattern modeling is that LP models lead to one unique solution for each sex-age group. There is no variability in the output result. By contrast, applying the LP model to individual diets allows the calculation of statistical distribution and provides more statistical power. However, previous studies in individual level LP modeling were based on 7-day food records from the nationally representative French enquete Individuelle Nationale des Consommations Alimentaires study, whereas the present NHANES database contained 1 day only.

The present results point to the importance of conducting feasibility studies before issuing dietary advice to the general population. Most dietary guidelines are still framed in terms of nutrients as opposed to foods and food groups. Given that foods contain mixtures of nutrients, nutrient-based guidelines may end up being incompatible with each other. In addition to nutrient constraints, LP models can take existing preferences and the economics of food choice into account.

## Figures and Tables

**Table:** MyPyramid food group characteristics of observed diets and optimized food patterns of nutrient recommendations and with an amount of sodium and potassium set to 1500 mg and 4700 mg, respectively, for men older than 51 years

	Observed food pattern	Modeled food pattern
Energy, kcal/d	1982	2000
Total weight, kg/d	1.4	1.8
MyPyramid food groups^a^		
Fruits, cup/d	1.3	3.8
Vegetable, cup/d	1.5	2.9
Total grains, oz eq/d	6.6	2.6
Whole grains, oz eq/d	0.9	1.2
Refined grains, oz eq/d	5.7	1.4
Meat and beans,^b^ oz eq/d	6.2	4.1
Milk,^c^ cup/d	1.4	1.9
Oils, g/d	16.0	28.1
SoFAS, kcal/d	679	462

a Quantity equivalents for each food groups are as follows: fruits and vegetables—1 cup equivalent is 1 cup raw or cooked fruit or vegetable, 1 cup fruit or vegetable juice, 2 cups leafy salad greens (1 cup = 237 mL); grains—1 oz equivalent is 1/2 cup cooked rice, pasta, or cooked cereal; 1 oz dry pasta or rice; 1 slice bread; 1 small muffin (1 oz); 1 cup ready-to-eat cereal flakes (1 oz = 28 g); meat and beans—1 oz equivalent is 1 oz lean meat, poultry, or fish; 1 egg; 1/4 cup cooked dry beans or tofu; 1 tbsp peanut butter; 1/2 oz nuts or seeds (1 oz = 28 g); milk—1 cup equivalent is 1 cup milk or yogurt, 11/2 oz natural cheese such as cheddar cheese, or 2 oz of processed cheese (1 cup = 237 mL).

b This group also contained poultry, eggs, and fish.

c This group also contained milk products.
